# Electronic and Transport Properties of Strained and Unstrained Ge_2_Sb_2_Te_5_: A DFT Investigation

**DOI:** 10.3390/ma16145015

**Published:** 2023-07-15

**Authors:** Jing Tian, Weiliang Ma, Pascal Boulet, Marie-Christine Record

**Affiliations:** 1 MADIREL, Department of Chemistry, CNRS, Aix-Marseille University, 13013 Marseille, France; jing.tian@etu.univ-amu.fr (J.T.); weiliang.ma@etu.univ-amu.fr (W.M.); 2 IM2NP, Department of Chemistry, CNRS, Aix-Marseille University, 13013 Marseille, France; m-c.record@univ-amu.fr

**Keywords:** layered chalcogenides, thermoelectrics, strains, density functional theory, Boltzmann transport

## Abstract

In recent years, layered chalcogenides have attracted interest for their appealing thermoelectric properties. We investigated the Ge_2_Sb_2_Te_5_ compound in two different stacking sequences, named stacking 1 (S1) and stacking 2 (S2), wherein the Ge and Sb atomic positions can be interchanged in the structure. The compound unit cell, comprising nine atoms, is made of two layers separated by a gap. We show, using the quantum theory of atoms in molecules, that the bonding across the layers has characteristics of transit region bonding, though with a close resemblance to closed-shell bonding. Both S1 and S2 are shown to bear a similar small gap. The full determination of their thermoelectric properties, including the Seebeck coefficient, electrical conductivity and electronic and lattice thermal conductivities, was carried out by solving the Boltzmann transport equation. We show that stacking 1 exhibits a larger Seebeck coefficient and smaller electrical conductivity than stacking 2, which is related to their small electronic gap difference, and that S1 is more suitable for thermoelectric application than S2. Moreover, under certain conditions of temperature and doping level, it could be possible to use S1-Ge_2_Sb_2_Te_5_ as both a *p* and *n* leg in a thermoelectric converter. Under biaxial, tensile and compressive strains, we observe that the thermoelectric properties are improved for both S1 and S2. Furthermore, the increase in the power factor of S1 in the cross-plane direction, namely perpendicular to the gap between the layers, shows that strains can counteract the electronic transport hindrance due to the gap.

## 1. Introduction

The rise in electrical energy needs over the decades calls for conducting deep investigations to improve storage and conversion systems. The discovery of efficient materials is one of the cornerstones that should allow for meeting the challenge of energy demand. Among the means to produce electrical power, both photovoltaic and thermoelectric conversions have regained strong interest. But both suffer from low conversion efficiency so far. Heat-to-electricity conversion has been known for more than two centuries, as this phenomenon was discovered, but remained unexplained at that time, in 1794 by Volta [1] and then rediscovered in 1821 by Seebeck [2], though erroneously interpreted by him as a manifestation of a magnetic field. Ioffe’s work in the early 1960s [3] has done much to popularize the use of semiconductors as potential thermoelectric materials, and thermoelectric modules have found a niche usage in spacecrafts [4]. More recently, thermoelectric materials have been implemented for heat-to-electricity conversion in cars’ exhaust-gas systems [5]. However, thermoelectricity has hardly found its way to large-scale deployment due to the lack of semiconductor materials with a figure of merit ZT greater than 2. One reason for this is that the figure of merit combines properties that counteract one another, i.e., the Seebeck coefficient and electrical conductivity that both appear in the numerator of ZT, and the electrical conductivity and electronic thermal conductivity that contribute to the numerator and denominator, respectively. The ZT expression then reads $\frac{S^{2}\sigma }{\kappa_{e}+\kappa_{l}}T$, where S is the Seebeck coefficient, σ the electrical conductivity, κ_e_ the electronic thermal conductivity, κ_l_ the lattice thermal conductivity and T the temperature. Hence, a compromise must be found to optimize materials ZT. Various families of material compounds have been shown to bear high thermoelectric (TE) properties, e.g., skutterudites, silicides, Zintl compounds and clathrates [6,7]. In the present work, the Ge_2_Sb_2_Te_5_ layered chalcogenide compound was investigated. Although this compound is well known as a phase change material [8], it shares with thermoelectrics chemical elements that are known to bring interesting TE properties, which legitimates the investigation of Ge_2_Sb_2_Te_5_ for its potential TE applications. In addition, the layered nature of Ge_2_Sb_2_Te_5_ is a beneficial feature for TE performance as it allows for decreasing lattice thermal conductivity. Very recently, Miao et al. [9] investigated Ge_2_Sb_2_Te_5_ using density functional theory approaches and found that, under certain conditions of temperature and carrier doping level, the figure of merit can reach 1.5. In their work, the ZT values were predicted by combining theoretical and experimental data. Ge_2_Sb_2_Te_5_ has also been investigated experimentally for its thermoelectric properties. It is recognized that this compound bears a decent ZT value, though it has a high electrical conductivity (due to high hole concentration) and hence a low Seebeck coefficient [10]. The substitution of In for Ge [10], Se for Te [11] and S/Se for Te [12] has shown the possibility of both increasing the Seebeck coefficient through the modulation of the density of states and decreasing the lattice thermal conductivity through mass disorder improvement, hence increasing ZT by 30% to almost 50%, reaching the values of 0.78 in Ge_1.85_In_0.15_Sb_2_Te_5_ at 700 K [10], 0.41 in Ge_2_Sb_2_Te_3.5_Se_1.5_ at 703 K [11] and 0.74 in Ge_2_Sb_2_Te_4.9_S_0_._1_ at 800 K [12]. According to these ZT values, it appears that Ge_2_Sb_2_Te_5_ bears similar thermoelectric performances to other well-known TE materials (skutterudites, Zintl phases, etc.).

In the present work, a fully ab initio approach was used to determine the TE properties. To date, the improvement in the TE properties of Ge_2_Sb_2_Te_5_ has been investigated using chemical substitution approaches, which have proven their efficiency, but not via induced strains on the material. Hence, we propose in our investigation to study the effects of strains on Ge_2_Sb_2_Te_5_ and demonstrate the possibility of improving the TE properties through the band engineering approach.

## 2. Computational Approaches

Density functional theory [13,14] (DFT) was used throughout to calculate the electronic properties of Ge_2_Sb_2_Te_5_. Local and generalized-gradient approximations were implemented with various exchange–correlation functionals (PZ [15], WC [16], PBE [17], PBEsol [18]) as well as the hybrid HSE06 [19] one to investigate its structural properties. For the subsequent TE property calculations, the WC functional was selected. The spin–orbit coupling was accounted for in these calculations for all the atoms. The Brillouin zone (BZ) was sampled with the *k*-meshes 12 × 12 × 2, 18 × 18 × 4 and 64 × 64 × 14 for structural optimizations, SCF calculation and transport property calculation, respectively. The total energy and atomic force convergence thresholds were defined as 0.136 meV and 0.257 meV.Å^−1^, respectively. The cutoff energy for the core–valence separation was fixed at −6.0 Ry. The *R*_MT_*K*_max_ value was set to 9.0 where *R*_MT_ is the minimum LAPW radius and *K*_max_ is the largest *k* vector in the Brillouin zone for the plane-wave cutoff. The radius of muffin tin (RMT) used for Ge, Sb and Te atoms in this work was set to 2.5 Å. The Wien2k package was used for these calculations [20,21]. 

The transport properties (Seebeck coefficient, electrical conductivity and electronic thermal conductivity) were determined using the Boltzmann transport equation (BTE) approach, as implemented in the BoltzTrap2 program [22]. The relaxation time of the electrons was calculated by applying the deformation potential theory [23,24].

The dynamic properties were calculated by means of the DFPT method by combining the Quantum-ESPRESSO package [25] and the Phono3py program [26,27]. A plane-wave energy cutoff of 70 Ry (952 eV) was employed. Total energies were minimized, with a convergence criterium of 10^−7^ Ry and a total force threshold of 10^−4^ Ry/bohr. A supercell of 2 × 2 × 2 was considered, which consists of a total of 72 atoms for Ge_2_Sb_2_Te_5_, with 4 × 4 × 1 *q*-mesh sampling. In subsequent postprocessing, phonon lifetimes were sampled using a finer 19 × 19 × 5 mesh.

## 3. Results and Discussion

In this section, we present and interpret the results of this investigation concerning the structural, electronic and transport features of the stacking 1 and stacking 2 Ge_2_Sb_2_Te_5_ compounds, both with and without applied strains. 

### 3.1. Unstrained Ge_2_Sb_2_Te_5_ Compounds

(a) Electronic Transport

**Figure 1:**
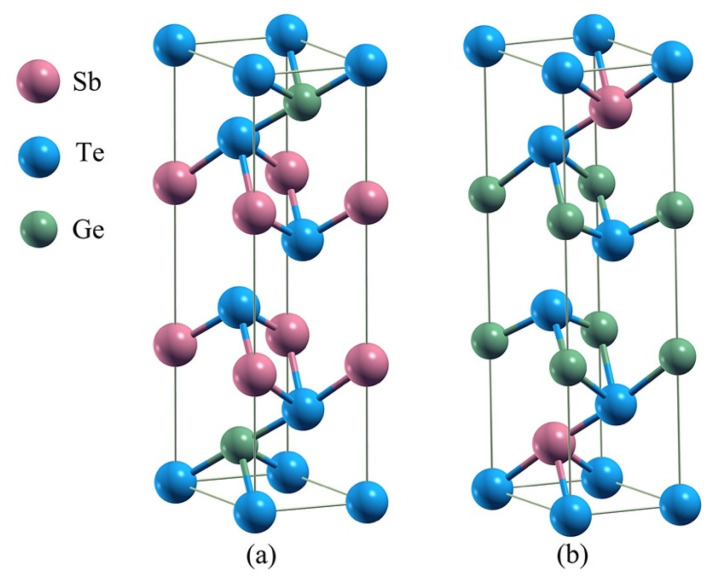
Hexagonal conventional cell representation of stacking 1 (**a**) and stacking 2 (**b**) polymorphs of Ge_2_Sb_2_Te_5_.

**Figure 2:**
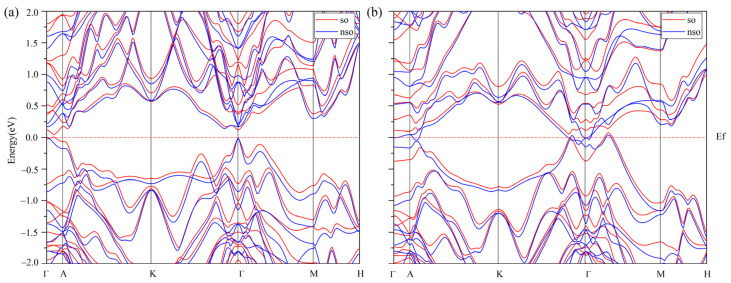
Electronic band structures in the Brillouin zone of the Ge_2_Sb_2_Te_5_ compound with stacking 1 (**a**) and stacking 2 (**b**) calculated with the WC functional with spin–orbit coupling (red) and without (blue). The energies are scaled using the Fermi energy (E_f_).

**Figure 3:**
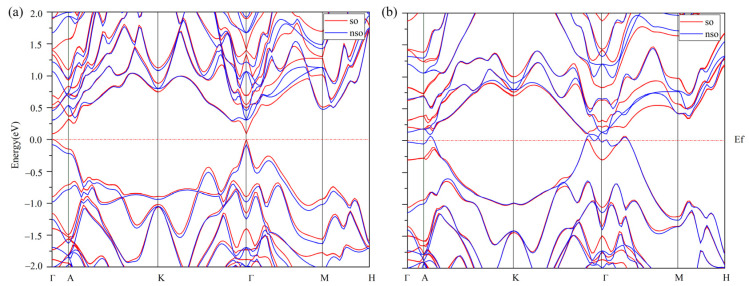
Electronic band structures in the Brillouin zone of the Ge_2_Sb_2_Te_5_ compound with stacking 1 (**a**) and stacking 2 (**b**) calculated with the HSE06 hybrid functional with spin–orbit coupling (red) and without (blue). The energies are scaled using the Fermi energy (E_f_).

The layered Ge_2_Sb_2_Te_5_ compounds crystallize in the rhombohedral Bravais system and belong to the space group number 164 ($P\bar{3}m1$). A hexagonal representation (conventional cell) of the stacking 1 and stacking 2 sequences of Ge_2_Sb_2_Te_5_ is depicted in Figure 1. From experimental crystallographic data, the c/a ratio amounts to 4.080 [28] and 4.299 [29] for stacking 1 and 4.080 [28] and 4.038 [30] for stacking 2 (Table 1). Averaged over all the functionals used, our results for the c/a ratio deviate by 0.6% and 5.6% for stacking 1 compared with Ref. [28] and Ref. [29], respectively, and by 2.3% and 4.3% for stacking 2, respectively. The lattice parameters are reasonably well reproduced by the functionals (Table 1), with average deviations for stacking 1 of 0.9% and 0.8% on *a* with respect to Ref. [28] and Ref. [29] and of 1.2% and 5.6% on *c*. For stacking 2, these deviations amount to 0.7% and 0.8% on *a* and 2.6% and 3.4% on *c*. As it turns out, the van der Waals-corrected PBE functional overestimates both the *a* and *c* parameters; therefore, considering the good performance of WC to reproduce the lattice parameters, this functional was chosen as the GGA one for further investigations. Nonetheless, our estimated band gap energies, including spin–orbit coupling or its absence, are seemingly underestimated with respect to the experimental findings, a feature that holds true for the hybrid HSE06 functional (Table 2). In any case, stacking 1 (S1) is found to be a small gap semiconductor (SC) while stacking 2 (S2) is a metal with the WC functional, whereas HSE06 describes S1 as small gap SC either with or without SOC, and S2 is either a metal without SOC or a small gap SC with SOC. The SOC interaction noticeably decreases the band gap energy of S1, but one does not observe specific features of SOC such as band splitting (Figure 2 and Figure 3). It is obvious that both the SOC and the exact exchange bring significant changes in the features of the electronic structures of S1 and S2 (Table 3). Assuming that HSE06-SOC is the most reliable approximation, one can summarize the results as follows: (1) both S1 and S2 are small gap SCs, (2) the gap of S1-Ge_2_Sb_2_Te_5_ is a direct one at Γ and that of S2-Ge_2_Sb_2_Te_5_ is an indirect one and (3) the energy gap of S1 is larger than that of S2. We note in passing that the WC-SOC approach also yields a direct band gap, though at a different *k*-point in the Brillouin zone. As transport property calculations are quite demanding in regard to Brillouin zone *k*-point sampling, the investigation of thermoelectric properties is intractable with the HSE06 functional, and we resorted to the WC+SOC one. 

The holes’ and electrons’ effective masses are, among other properties, important characteristics for electronic transport, and relate to the inverse curvature of the highest valence band and the lowest conduction band, respectively. The curvature of the valence and conduction bands of S1 in the *ab* plane is smaller than that in the *c* direction (Table 4), leading to larger mobility of the charge carriers. In the simple picture of the Drude model, the electrical conductivity ($\sigma =ne^{2}\tau /m^{*}$) should then be higher in the basal plane of the structure. The same feature is observed for stacking S2 of Ge_2_Sb_2_Te_5_.

**Figure 4:**
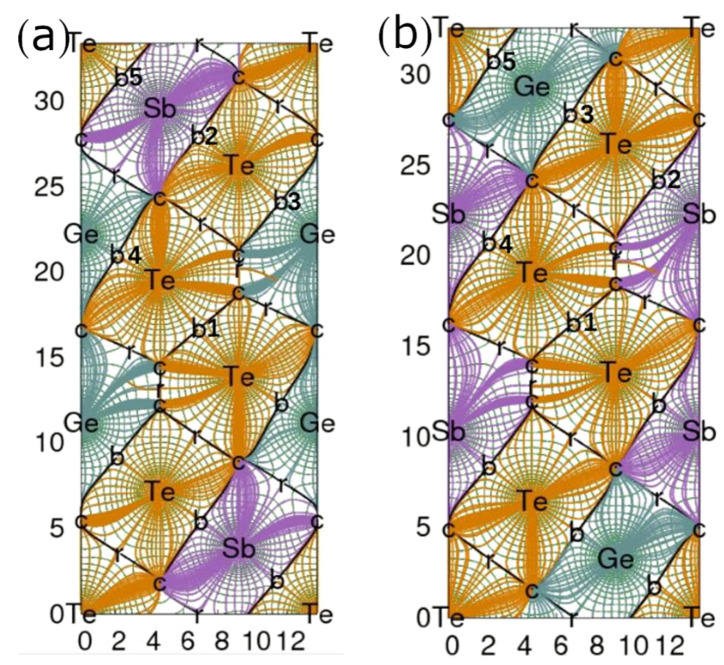
Electron density gradient of S1-Ge_2_Sb_2_Te_5_ (**a**) and S2-Ge_2_Sb_2_Te_5_ (**b**) plotted in the (110) plane. b: bond critical point; r: ring critical point; c: cage critical point.

**Figure 5:**
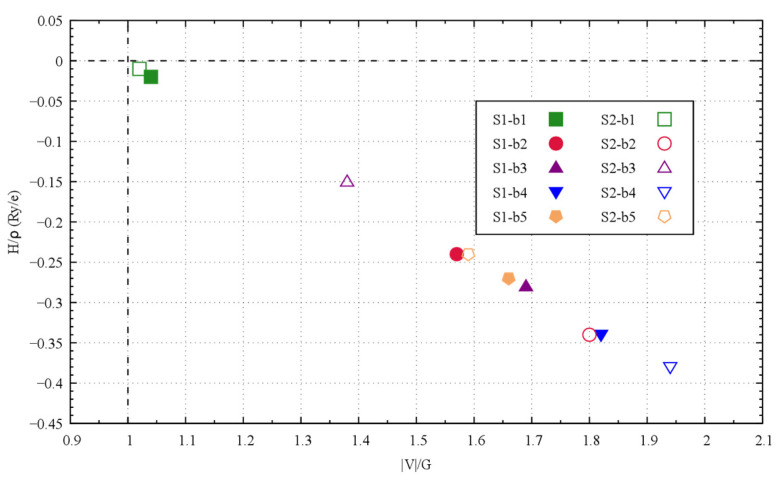
Bond degree vs. |V|/G ratio of the bonding interactions in S1- and S2-Sb_2_Te_2_Ge_5_.

The bonding structures of S1- and S2-Ge_2_Sb_2_Te_5_ can be characterized by the electron density gradient (Figure 4). The zero-gradient lines delineating the so-called atomic basins allow for visualizing the atomic volumes, which are noticeably larger for Te and Ge than for Sb and are far from spherical in shape. The line of maximum gradient joining two atoms and passing through a bond critical point characterizes a bond path. In this respect, the Te atoms next to and on either side of the interlayer space are connected by a bond path (BCP, labeled b1 in Figure 4). The interlayer interaction is characterized by a low density, a slightly negative total energy density and a negative, though small, bond degree (H/ρ) (Table 5). The positive value of the electron density Laplacian (∇^2^ρ) underscores the tendency of the electron density to escape from the BCP region. Strictly speaking, the interlayer bonding interaction corresponds to transit region bonding, as defined by Espinosa [36], with the characteristics 1< |V|/G < 2, H/ρ < 0 and ∇^2^ρ > 0, although this interaction is very close to that corresponding to the closed-shell region, which includes the van der Waals interactions. All the other bond interactions, labeled b2 to b5, are characteristic of the transit region bonding interaction (Table 5). The variety of bonds in S1- and S2-Ge_2_Sb_2_Te_5_ structures is depicted in Figure 5, which represents the bond degree with respect to the |V|/G ratio [37]. The nearly-closed-shell S1-b1 and S2-b1 interactions are clearly well separated from the other bonding interactions, bearing both small bond degrees and |V|/G ratios, which is a sign of rather weak interactions. Interestingly, all the bonding interactions in these compounds share the same rigidity, as defined by Yang et al. [37,38], with G/ρ ≈ 0.41 Ha/e, which corresponds to the slope of the line (Figure 5). The weak interlayer interaction, characterized by a low bond degree and low electron density having a tendency to escape from this region, suggests a strong resistance to electron conduction along the *c*-axis of the compounds; hence, a low electrical conductivity σ_zz_ and a high Seebeck coefficient are expected in this direction.

**Figure 6:**
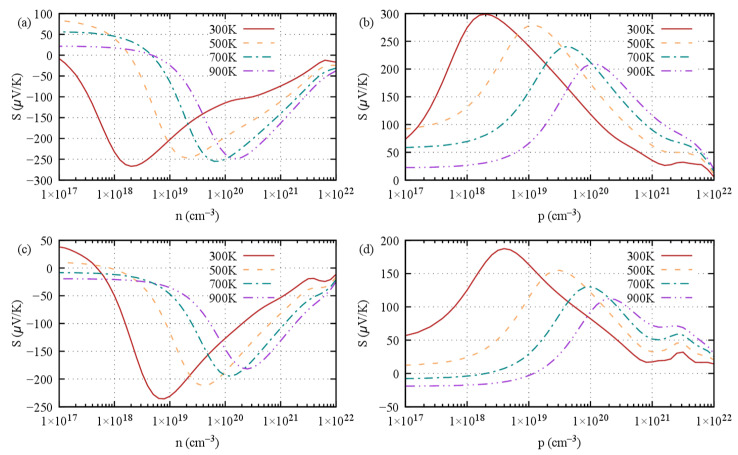
Seebeck coefficient at 300 K, 500 K, 700 K and 900 K of Ge_2_Sb_2_Te_5_ with (**a**,**b**) stacking 1 and (**c**,**d**) stacking 2 with respect to (**a**,**c**) *n*-type doping level and (**b**,**d**) *p*-type doping level.

The average Seebeck coefficient (Figure 6), as calculated with the WC-SOC and BTE approach, shows a broad peak for both *n*- and *p*-doped S1- and S2-Ge_2_Sb_2_Te_5_, the maximum of which is being shifted towards a higher doping level as the temperature increases. The magnitude of the maxima for the S1 stacking is larger (in absolute value) than that of the S2 stacking, which is a consequence of the larger gap of S1 compared with that of S2. Overall, a high maximum Seebeck coefficient is found that ranges between 120 μV K^−1^ and 300 μV K^−1^, in absolute values. As expected from the bonding analysis, the cross-plane Seebeck coefficient is noticeably higher than that in the *ab* plane (see Figures S1 and S2 in the supplemental data), especially for stacking 1 at temperatures above 300 K. This confirms that the gap between the layers hinders electron conduction, hence improving S in this direction. 

As the linearized Boltzmann transport equation leads to τ-scaled electrical conductivity σ/*τ*, where *τ* is the carrier relaxation time, the values of *τ* for both electrons and holes were determined using the deformation potential theory [23,24]. According to this theory, the relaxation time can be determined from the following relation:

(1) $$\tau_{i}=\frac{2\sqrt{2\pi }\hbar^{4}}{3{\left(k_{B}T\right)}^{3/2}}\times \frac{C_{ii}}{m_{i}^{*3/2}E_{d}^{i2}}$$

where $C_{ii}$ is the elastic constant in the *i* direction, $m_{i}^{*}$ is the carrier effective mass and $E_{d}^{i}$ is the valence (for holes) or conduction (for electrons) band orbital energy variation when the lattice is subjected to a distortion along the direction i:

(2) $$E_{d}^{i}=\frac{dE}{d\left(l^{i}/l_{0}^{i}\right)}$$

*l*_0_ being the undistorted lattice parameter. The values of *τ* calculated at 300 K, 500 K, 700 K and 900 K are gathered in Table 6. Overall, the relaxation times of the electrons lie in the range 10^–13^–10^–11^ s. The scattering time in the *z* direction is shorter than that in the *ab* plane, irrespective of the stacking type and temperature, highlighting the increased scattering by the interlayer interface. It is hence expected that there is a larger in-plane electrical conductivity and a smaller cross-plane one.

**Figure 7:**
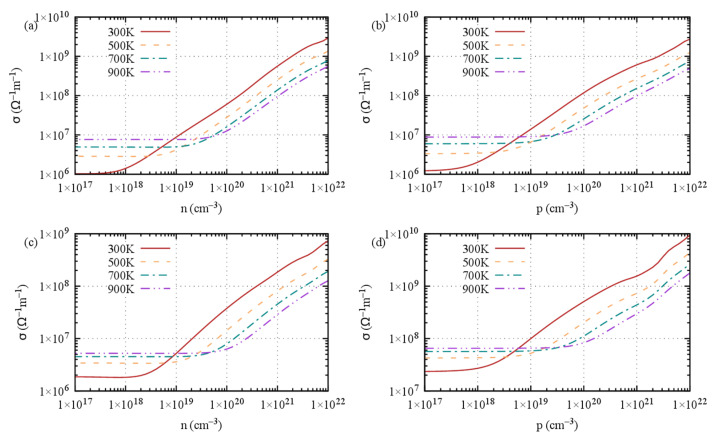
Electrical conductivity at 300 K, 500 K, 700 K and 900 K of Ge_2_Sb_2_Te_5_ with (**a**,**b**) stacking 1 and (**c**,**d**) stacking 2 with respect to (**a**,**c**) *n*-type doping level and (**b**,**d**) *p*-type doping level.

The average electrical conductivity (Figure 7) shows similar trends for both S1 and S2, irrespective of the type of doping; a plateau is observed up to 10^18^–10^19^ carriers/cm^−3^ and then a steady increase when the compound becomes a degenerate semiconductor. More interesting are the in-plane and cross-plane features of the electrical conductivity, in line with what we expected from the bonding analysis (Figures S3 and S4). Indeed, the cross-plane electrical conductivity is substantially lower by about one order of magnitude for stacking 1, irrespective of the *n*- or *p*-doping type, confirming the barrier-like behavior of the gap between the layers. It is obvious that the gap behavior has much more impact on the electrical conductivity than on the Seebeck coefficient. Assuming the Mott formula for S holds (S∝dln(σ)), this behavior might be explained by the fact that the large effect on σ is damped by the log function in S. 

**Figure 8:**
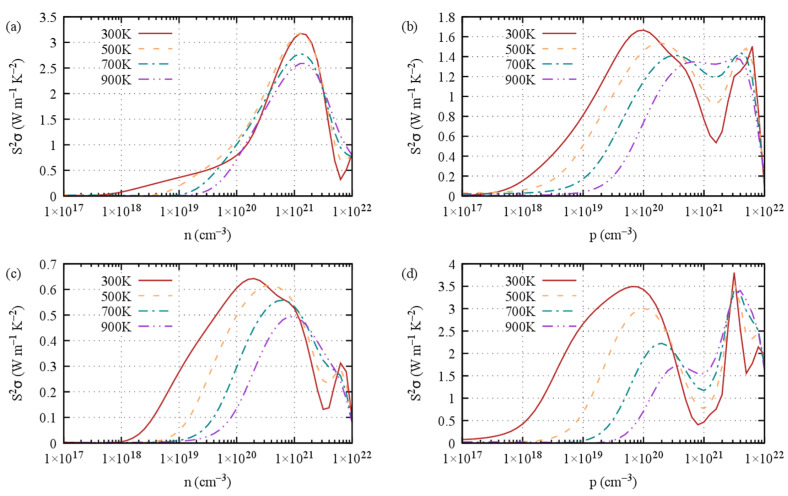
Power factor (S^2^*σ*) at 300 K, 500 K, 700 K and 900 K of Ge_2_Sb_2_Te_5_ with (**a**,**b**) stacking 1 and (**c**,**d**) stacking 2 with respect to (**a**,**c**) *n*-type doping level and (**b**,**d**) *p*-type doping level.

Ultimately, the power factor characterizes the electrical transport properties of a thermoelectric material. The power factor (PF) reads S^2^*σ*. For S1, the PF exhibits a wide peak at large carriers’ concentrations of about 10^21^ cm^−3^ for *n*-type doping (Figure 8a) and 10^20^ cm^−3^ for *p*-type doping (Figure 8b). As it turns out, the overall power factor of S1 Ge_2_Sb_2_Te_5_ is mostly contributed by the in-plane PF, both for *n*-type (Figure S5a) and *p*-type doping (Figure S5b). The cross-plane PF (Figures S6a and S6b) is much weaker than the in-plane one, which can be related to the much smaller electrical conductivity that overkills the otherwise higher Seebeck coefficient.

(b) Thermal Transport

**Figure 9:**
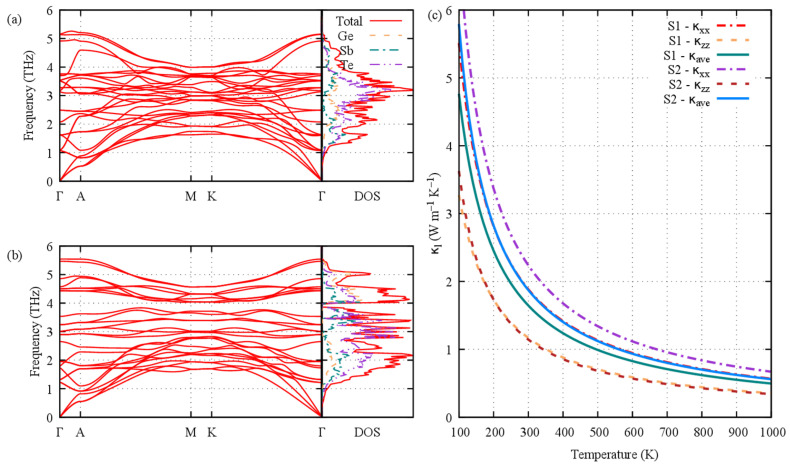
Phonon spectrum and total and projected phonon density of states of (**a**) stacking 1 and (**b**) stacking 2 at equilibrium. (**c**) Calculated lattice thermal conductivity of stackings 1 and 2. The evolution is plotted for the in-layer a-axis, cross-layer c-axis and average values.

Using the DFPT approach, the anharmonic force constants were calculated for the two stacking sequences in the *ab* plane and along the *c*-axis, and the corresponding phonon spectrum and DOS are presented in Figure 9a,b. We observed a small frequency gap between 3.9 and 4.0 THz for stacking 2, which is comparable to but smaller in extent than the gap observed in the Pb_2_Bi_2_Te_5_ compound [39]. The DOS shows the prominent contribution of the Te atoms over the whole spectrum range, especially in the acoustic and low-energy (1.5–3 THz) optical phonons. The Sb contribution is appreciable except between 2.2 and 3 THz, and the Ge contribution is appreciable above 2.2 THz only. The lattice thermal conductivity *κ*_l_ was evaluated using the single-mode relaxation time approximation (RTA):

(3) $$\kappa_{l}=\frac{1}{NV_{0}}\sum_{\lambda }C_{\lambda }\nu_{\lambda }⨂\nu_{\lambda }\tau_{\lambda }$$

where *N* is the number of *q*-points, $V_{0}$ is the volume of the unit cell and $C_{\lambda }$, $\nu_{\lambda }$ and $\tau_{\lambda }$ are heat capacity, group velocity and phonon lifetime, respectively. 

**Figure 10:**
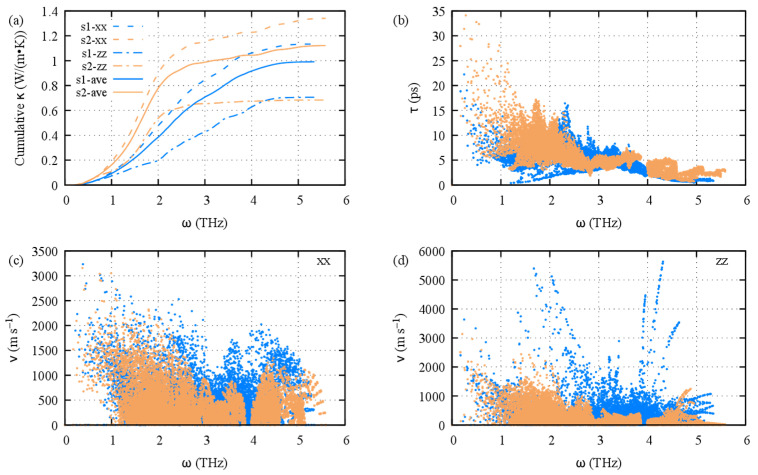
Calculated cumulative lattice thermal conductivity for Ge_2_Sb_2_Te_5_ stacking 1 (blue) and stacking 2 (orange) with RTA (**a**). Phonon lifetimes for S1 and S2 (**b**). In-layer group velocity (**c**) and cross-layer group velocity (**d**) for both S1 and S2. Results obtained at 500 K.

The calculated lattice thermal conductivity for the two stacking sequences in the *ab* plane ($\kappa_{xx}$) and along the *c*-axis $\kappa_{zz}$ and the average thermal conductivity $\kappa_{l,ave}=(2\times \kappa_{xx}+\kappa_{zz})/3$ are shown in Figure 9c. Overall, the lattice thermal conductivity is lower for S1 than for S2, which is caused by the smaller in-plane contribution of S1 compared with that of S2; the cross-plane contribution is the same for both sequences. Therefore, in S2, one can state that the frequency gap shows only marginal effects on the total lattice thermal conductivity; a very small plateau is observed in $\kappa_{xx}$ between 3.9 and 4.0 THz, which is slightly reflected in $\kappa_{l,\;ave}$ (Figure 10a). Interestingly, in stacking 2 the cross-plane lattice thermal conductivity exhibits a wide plateau at 0.7 W/(m·K) from 2.5 THz onward, but this behavior is not strong enough to counterbalance the steep increase in the in-plane contribution at low energy (acoustic phonons) that, by far, surpasses the S1 $\kappa_{xx}$ and $\kappa_{zz}$ contributions. The wide plateau of $\kappa_{zz}$ for S2 could be related to the small velocities of the optical phonons, especially above 3 THz (Figure 10d). Clearly, the overall smaller lattice thermal conductivity of S1 is caused by the smaller acoustic phonon scattering times (Figure 10b). Indeed, although we observe that the velocities of the acoustic phonons of S1 are about the same as those of S2 and that the velocities of the optical phonons of S1 are much higher than those of stacking 2, the phonon lifetimes of S2 are much longer than those of S1 in the low-frequency range of 0–3 THz (acoustic and optical modes), which contributes to 70% and 91% of the total $\kappa_{l}$ of S1 and S2, respectively. In a sense, the phonons of S2 transport heat more slowly but more surely, hence more efficiently.

It has been stated in the literature [40,41] that low lattice thermal conductivity could be related to a strong coupling between low-energy optical phonons and acoustic phonons, leading to avoided crossing in the phonon band structure. As a tentative explanation of the lower thermal conductivity of S1 compared with that of S2, one may invoke such a larger coupling in S1 than in S2. Several avoided band crossings between acoustic and optical phonons may possibly appear (Figure 9a) in S1, e.g., at the A k-point around 1 THz, and on the K-Γ line (at ξ≈1/3 along this line around 2 THz and ξ≈3/4 around 1.2 THz), the former coupling being seemingly much weaker and the latter one being absent in S2 (Figure 9b). The strong phonon coupling has been attributed to the distortion of the structure around the central atom, namely the deviation, for instance, from an octahedral or a tetrahedral symmetry: the larger the deviation, the larger the mode anharmonicity. However, this effect cannot be evoked for Ge_2_Sb_2_Te_5_ as the larger deviation of the central atom (Sb in our case) from the center of the octahedral cage (see Table S1) is observed for the S2 structure for which the lattice thermal conductivity is higher than that of S1. Hence, the metallic state of S2 (in the limit of the WC functional used here) is likely the reason for the higher lattice thermal conductivity of S2.

(c) Figure of Merit

**Figure 11:**
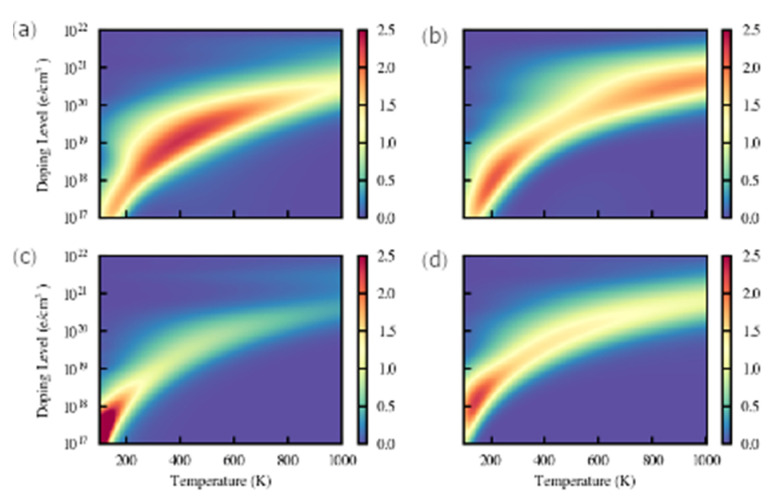
Calculated figures of merit ZT of Ge_2_Sb_2_Te_5_ stacking 1 (**a**,**b**) and stacking 2 (**c**,**d**). Both *p*-type (**a**,**c**) and *n*-type (**b**,**d**) doping are considered.

The figure of merit ZT can be evaluated from the power factor and the lattice thermal conductivities. The results of ZT as a function of carrier concentrations and temperatures for stackings 1 and 2 are shown in Figure 11. The ranges of conditions to obtain the maximum ZT for stacking 1 are 200–700 K/10^18^–10^20^ holes/cm^3^ for *p*-type doping (Figure 11a), and 150–300 K/10^17^–10^19^ electrons/cm^3^ and 900–1000 K/10^20^–10^21^ electrons/cm^3^ for *n*-type doping (Figure 11b), hence the suitable region for *n*-type doping splits into two parts, namely one located at a high temperature and high doping level and the other one at a low temperature and low doping level. The favorable working temperature is rather wide for the *p*-type, but for high temperatures *n*-type doping is more suitable. Interestingly, S1-Ge_2_Sb_2_Te_5_ could be used both as a *p* and *n* leg in a thermoelectric converter at high temperatures (about 800 K) where their ZT values are predicted to be similar (about 1.7). The highest value is 2.3 for stacking 1 with *p*-type doping. By contrast, the suitable conditions for stacking 2 to obtain high ZT are unfavorable for applications as they correspond to temperatures well below the room temperature, irrespective of the doping type.

### 3.2. Electronic Transport Properties of the Ge_2_Sb_2_Te_5_ Compounds under Tensile and Compressive Strains

As the calculation of the lattice thermal conductivity is still a computationally heavy task, it was not possible to calculate $\kappa_{l}$ for S1 and S2 for all the applied strains. Therefore, we focus in this part on the electronic transport properties and more specifically on the Seebeck coefficient and the power factor.

**Figure 12:**
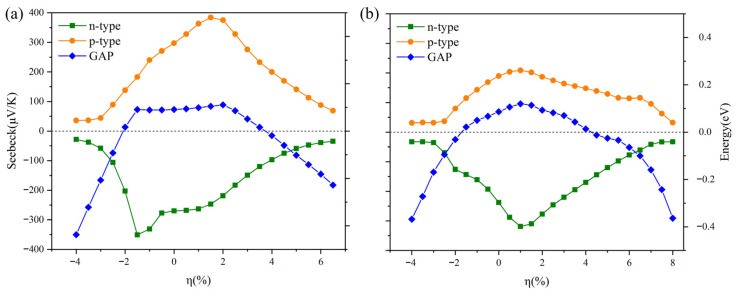
Energy gap and Seebeck coefficient at 300 K vs. compressive (η < 0) and tensile (η > 0) biaxial strains applied to (**a**) S1 and (**b**) S2 Ge_2_Sb_2_Te_5_ compounds.

Biaxial, compressive (η < 0) and tensile (η > 0) strains were applied to the S1- and S2-Ge_2_Sb_2_Te_5_ compounds along the basal *ab* plane. As expected, the electronic structure of the compounds is affected by the strains, which is manifested by the change in the energy gap that either slightly widens or strongly shrinks up to the point where the compounds become metallic (Figure 12). A concomitant evolution of the Seebeck coefficient is observed as it reaches a maximum value when the gap is largest; this is particularly evident for the S2 stacking (Figure 12b) for which the maximum gap comes up at +1% tensile strain that correlates exactly with the maximum of S for both *n-* and *p*-doped compounds. For S1, the behavior is somewhat different. We note first that the gap is almost constant in the range of −1.75% to +2% strains. At these extremes, the Seebeck coefficient reaches a maximum of about 380 μV K^−1^ under tensile strain for *p*-type doping and a maximum of about |-350| μV K^−1^ under compressive strain for *n*-type doping. Clearly, the Seebeck coefficient can be noticeably improved by applying biaxial strains, which could be achieved experimentally by depositing Ge_2_Sb_2_Te_5_ on a suitably selected support. 

**Figure 13:**
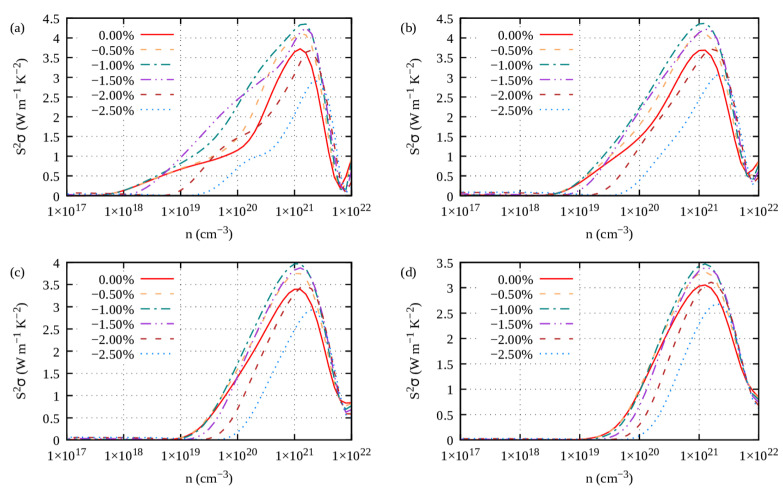
*ab* plane power factor of S1-Ge_2_Sb_2_Te_5_ calculated under compressive strains (η < 0) for electron carriers and temperatures of (**a**) 300 K, (**b**) 500 K, (**c**) 700 K and (**d**) 900 K.

**Figure 14:**
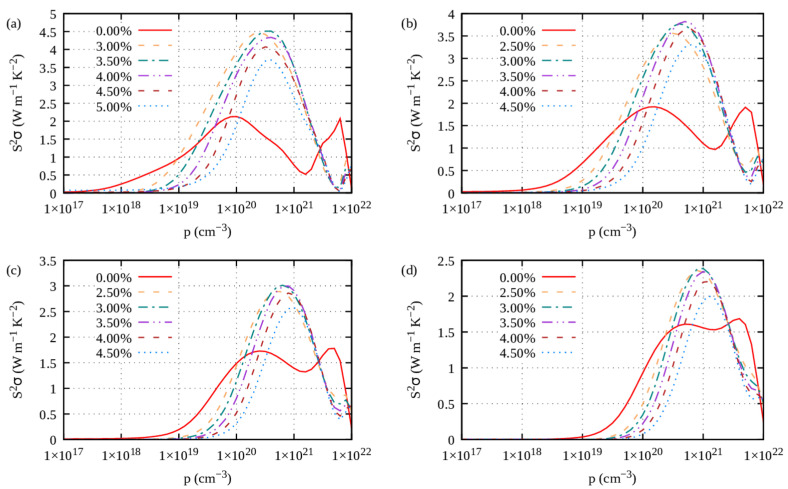
*ab* plane power factor of S1-Ge_2_Sb_2_Te_5_ calculated under tensile strains (η > 0) for hole carriers and temperatures of (**a**) 300 K, (**b**) 500 K, (**c**) 700 K and (**d**) 900 K.

**Figure 15:**
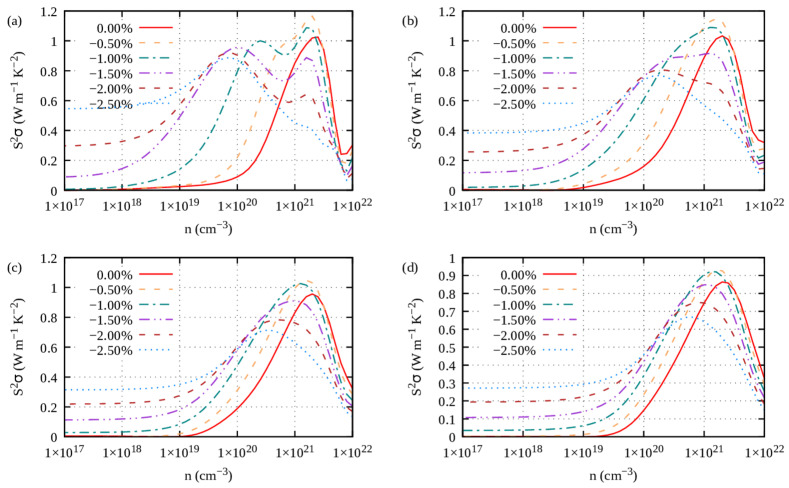
*z* direction power factor of S1-Ge_2_Sb_2_Te_5_ calculated under compressive strains (η < 0) for electron carriers and temperatures of (**a**) 300 K, (**b**) 500 K, (**c**) 700 K and (**d**) 900 K.

**Figure 16:**
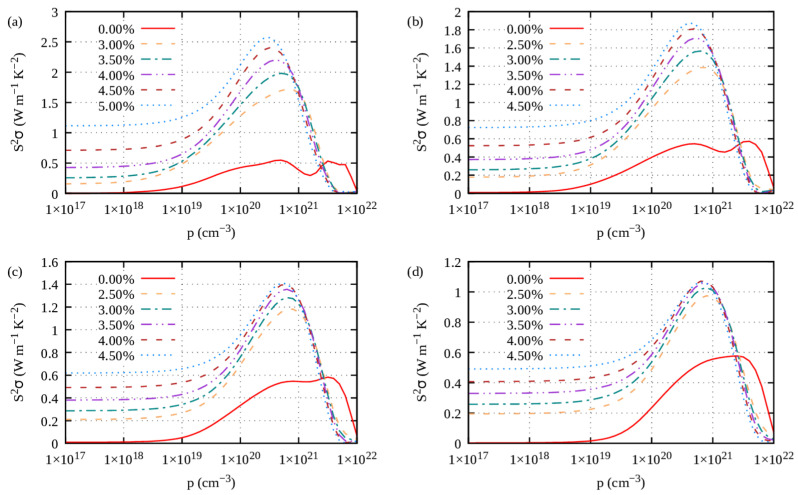
*z* direction power factor of S1-Ge_2_Sb_2_Te_5_ calculated under tensile strains (η > 0) for hole carriers and temperatures of (**a**) 300 K, (**b**) 500 K, (**c**) 700 K and (**d**) 900 K.

Under tensile strains with electron carriers and compressive strains with hole carriers, the power factor (PF) of the S1-Ge_2_Sb_2_Te_5_ compound is improved (Figure 13, Figure 14, Figure 15 and Figure 16), irrespective of the direction (*ab* plane or *z* direction). We note here that the electron scattering times are those of the unstrained structure. Still, noticeable differences can be underlined. The improvement is small when S1 is doped with electrons, whereas it is impressively increased when it is doped with holes. This behavior holds true whether transport occurs in-plane or cross-plane; thus, strains can counteract the fact that the cross-plane gap between the layers hinders electronic transport. Hence, on average, under compressive strains and with hole doping, S1 exhibits an excellent power factor ranging from 2 to 4 W m^−1^ K^−2^ between 700 K and 300 K (Figure S8). By contrast, under tensile strains and with electron doping, S1 shows a similar PF to that when unstrained (Figure S9). Overall, the same behavior is observed for the S2-Ge_2_Sb_2_Te_5_ compound when strained (Figures S10–S15).

## 4. Conclusions

In this paper, a thorough investigation of the electronic and thermoelectric properties of the Ge_2_Sb_2_Te_5_ compound is presented. The two different stacking sequences, namely stacking 1 (S1) and stacking 2 (S2), for which Ge and Sb can be swapped, were investigated by combining density functional theory, the quantum theory of atoms in molecules (QTAIM) and the Boltzmann transport theory. At the hybrid functional level including spin–orbit interaction, both S1 and S2 are shown to be semiconducting materials with close, small gap energies (0.090 eV and 0.086 eV, respectively). S1 bears a direct gap whereas S2 has an indirect one. From the QTAIM theory, the analysis of the electron density Laplacian and energy densities at the bond critical points allows us to conclude that the interlayer bonding interaction is of transit region nature, though sharing features of closed-shell interaction. The thermoelectric properties of both unstrained and strained S1 and S2 stackings were calculated. It was found that the S1 stacking is more suitable for use in a thermogenerator, both as a *p* and an *n* leg, as it exhibits high ZT values under certain ranges of temperatures and doping levels. Under strains, we observe that the thermoelectric properties of both S1 and S2 are improved. The improvement is more obvious when the material is *p*-doped under compressive strains than when it is *n*-doped under tensile strains. Remarkably, the strains are amenable to preventing the cross-plane electronic transport hindrance caused by the interlayer gap.

## Figures and Tables

**Table 1:** Lattice parameters (in Å) and atomic positions (in crystallographic coordinates) of the stacking 1 and stacking 2 Ge_2_Sb_2_Te_5_. Only the *z* crystallographic coordinates are provided, as the *x* and *y* ones are fixed by the crystal symmetry.

		LDA	WC	PBE	PBEsol	PBE-vdw	Exp.
Ge_2_Sb_2_Te_5_ S1	*a*	4.20	4.24	4.30	4.23	4.28	4.225 [28] 4.25 [29]
	*c*	17.09	16.92	17.51	17.23	17.47	17.239 [28] 18.27 [29]
	Ge	0.0998	0.1006	0.0988	0.0995	0.0997	
	Te2	0.1974	0.1990	0.1953	0.1968	0.1971	
	Sb	0.3153	0.3178	0.3113	0.3141	0.3142	
	Te3	0.4161	0.4198	0.4091	0.4143	0.4132	
Ge_2_Sb_2_Te_5_ S2	*a*	4.18	4.22	4.27	4.21	4.25	4.225 [28] 4.20 [30]
	*c*	17.49	17.13	18.01	17.65	17.90	17.239 [28] 16.96 [30]
	Ge	0.3353	0.3387	0.3329	0.3337	0.3361	
	Te2	0.2151	0.2188	0.2094	0.2139	0.2114	
	Sb	0.1150	0.1167	0.1124	0.1143	0.1135	
	Te3	0.4161	0.4213	0.4116	0.4143	0.4158	

**Table 2:** Band gap energies (eV) of stacking 1 and stacking 2 Ge_2_Sb_2_Te_5_ compounds with and without spin–orbit coupling (SOC and non-SOC).

	WC	HSE06	Exp.	Other Calculations
S1 (Non-SOC)	0.147	0.384	0.5 [31,32,33]	0.26 (PBE) [34], 0.3 (PBE-D2) [35], 0.49 (HSE06-D2) [35]
S1 (SOC)	0.039	0.090		
S2 (Non-SOC)	0	0.122		0 (PBE) [34]
S2 (SOC)	0	0.086		

**Table 3:** Effects of the spin–orbit coupling (SOC) and exact exchange interaction, through the HSE06 functional, on the band structures of stacking 1 and stacking 2 of Ge_2_Sb_2_Te_5_ as observed from Figure 2 and Figure 3.

	WC Band Gap Location or Closure		HSE06 Band Gap Location or Closure	
	Without SOC	With SOC	Without SOC	With SOC
S1	Opened, Γ → Γ-K path	Opened, Γ-A → Γ-A paths	Opened, Γ → Γ-K path	Opened, Γ → Γ
S2	Closed, A-K and Γ-K paths	Closed, Γ-M and Γ-K paths	Closed, A-K, Γ-K and Γ-M paths	Opened, Γ-M → Γ-K paths

**Table 4:** Relative effective masses (m* in unit of the electron rest mass) of holes and electrons and potential deformation energies (E_d_), calculated in the *c*-axis direction and *ab* plane for stacking 1 and stacking 2 of Ge_2_Sb_2_Te_5_ with the WC+SOC approach.

Structure	Charge	m*		E_d_ (eV)	
		c	a,b	c	a,b
Ge_2_Sb_2_Te_5_ S1	holes	−0.0974	−0.0187	7.01	13.39
Ge_2_Sb_2_Te_5_ S1	electrons	0.0797	0.0175	10.09	14.88
Ge_2_Sb_2_Te_5_ S2	holes	−0.0215	−0.0065	6.92	14.16
Ge_2_Sb_2_Te_5_ S2	electrons	0.0580	0.0448	9.52	12.66

**Table 5:** Bond critical point (BCP) properties of S1- and S2-Sb_2_Te_2_Ge_5_ calculated from the topological analysis of the electron density according to the quantum theory of atoms in molecules. b1 to b5: bond critical point (see Figure 4); r_1_ and r_2_: distances (in pm) between the BCP and the closest atomic nuclei; θ: angle (in degrees) between the BCP and the two nuclei it connects (the BCP being the apex); ρ: electron density at the BCP (in electron/Å^3^); ∇^2^ρ: electron density Laplacian at the BCP (in electron/Å^5^); G, V and H: kinetic, potential and total energy density at the BCP (in Ha/Å^3^); H/ρ: bond degree (in Ha/electron).

BCP	r_1_	r_2_	r_1_/r_2_	θ	ρ	∇^2^ρ	G	V	H	|V|/G	H/ρ
S1-b1	190.8	190.8	1.000	180.00	1.145	1.779	0.46	−0.48	−0.02	1.04	−0.02
S1-b2	163.7	153.9	1.063	179.76	3.301	2.343	1.37	−2.15	−0.78	1.57	−0.24
S1-b3	136.8	159.8	0.856	179.56	3.791	1.952	1.55	−2.62	−1.07	1.69	−0.28
S1-b4	155.5	144.0	1.080	179.41	4.608	1.399	1.93	−3.52	−1.58	1.82	−0.34
S1-b5	138.1	160.8	0.859	179.97	3.645	2.034	1.49	−2.47	−0.98	1.66	−0.27
S2-b1	192.6	192.6	1.000	180.00	1.089	1.717	0.44	−0.45	−0.01	1.02	−0.01
S2-b2	144.5	155.6	0.929	179.55	4.530	1.558	1.91	−3.44	−1.52	1.80	−0.34
S2-b3	170.7	152.9	1.116	177.53	2.368	2.370	0.96	−1.32	−0.36	1.38	−0.15
S2-b4	128.7	153.2	0.840	178.99	5.012	0.487	2.04	−3.95	−1.92	1.94	−0.38
S2-b5	153.3	163.0	0.941	179.96	3.384	2.299	1.40	−2.22	−0.82	1.59	−0.24

**Table 6:** Relaxation time (in s) of the electron (e) and hole (h) carriers in the *ab* plane and *z* direction for stackings S1 and S2 at 300 K, 500 K, 700 K and 900 K.

T (K)	Stacking	$\tau_{e}^{ab}$	$\tau_{e}^{z}$	$\tau_{h}^{ab}$	$\tau_{h}^{z}$
300	S1	6.70 × 10^–12^	1.25 × 10^–12^	7.49 × 10^–12^	1.92 × 10^–12^
	S2	2.08 × 10^–12^	1.98 × 10^–12^	3.00 × 10^–11^	1.66 × 10^–11^
500	S1	3.11 × 10^–12^	5.81 × 10^–13^	3.48 × 10^–12^	8.92 × 10^–13^
	S2	9.67 × 10^–13^	9.20 × 10^–13^	1.39 × 10^–11^	7.71 × 10^–12^
700	S1	1.88 × 10^–12^	3.51 × 10^–13^	2.10 × 10^–12^	5.39 × 10^–13^
	S2	5.84 × 10^–13^	5.56 × 10^–13^	8.42 × 10^–12^	4.66 × 10^–12^
900	S1	1.29 × 10^–12^	2.41 × 10^–13^	1.44 × 10^–12^	3.70 × 10^–13^
	S2	4.00 × 10^–13^	3.81 × 10^–13^	5.77 × 10^–12^	3.19 × 10^–12^

## Data Availability

Data are available upon request to the authors.

## Supplementary Materials

The following supporting information can be downloaded at https://www.mdpi.com/article/10.3390/ma16145015/s1: Figure S1: Seebeck coefficient in the ab plane of Ge_2_Sb_2_Te_5_ at 300 K, 500 K, 700 K and 900 K for (a,b) stacking 1 and (c,d) stacking 2 with respect to (a,c) *n*-type doping level and (b,d) *p*-type doping level; Figure S2: Seebeck coefficient in the cross-plane (c-axis direction) of Ge_2_Sb_2_Te_5_ at 300 K, 500 K, 700 K and 900 K for (a,b) stacking 1 and (c,d) stacking 2 with respect to (a,c) *n*-type doping level and (b,d) *p*-type doping level; Figure S3: Electrical conductivity in the ab plane of Ge_2_Sb_2_Te_5_ at 300 K, 500 K, 700 K and 900 K for (a,b) stacking 1 and (c,d) stacking 2 with respect to (a,c) *n*-type doping level and (b,d) *p*-type doping level; Figure S4: Electrical conductivity in the cross-plane (c-axis direction) of Ge_2_Sb_2_Te_5_ at 300 K, 500 K, 700 K and 900 K for (a,b) stacking 1 and (c,d) stacking 2 with respect to (a,c) *n*-type doping level and b,d) *p*-type doping level; Figure S5: Power factor (S^2^*σ*) in the ab plane of Ge_2_Sb_2_Te_5_ at 300 K, 500 K, 700 K and 900 K for (a,b) stacking 1 and (c,d) stacking 2 with respect to (a,c) *n*-type doping level and (b,d) *p*-type doping level; Figure S6: Power factor (S^2^*σ*) in the cross-plane (c-axis direction) of Ge_2_Sb_2_Te_5_ at 300 K, 500 K, 700 K and 900 K for (a,b) stacking 1 and (c,d) stacking 2 with respect to (a,c) *n*-type doping level and (b,d) *p*-type doping level; Figure S7: Deformed octahedral environment of (a) Ge in S1 stacking; (b) Sb in S2 stacking; Figure S8: Average power factor of *p*-doped S1-Ge_2_Sb_2_Te_5_ with respect to tensile strains at the temperatures of (a) 300 K, (b) 500 K, (c) 700 K and (d) 900 K; Figure S9: Average power factor of *n*-doped S1-Ge_2_Sb_2_Te_5_ with respect to compressive strains at the temperatures of (a) 300 K, (b) 500 K, (c) 700 K and (d) 900 K; Figure S10: *ab* plane power factor of S2-Ge_2_Sb_2_Te_5_ calculated under compressive strains (η < 0) for electron carriers and temperatures of (a) 300 K, (b) 500 K, (c) 700 K and (d) 900 K; Figure S11: *ab* plane power factor of S2-Ge_2_Sb_2_Te_5_ calculated under tensile strains (η > 0) for hole carriers and temperatures of (a) 300 K, (b) 500 K, (c) 700 K and (d) 900 K; Figure S12: *z* direction power factor of S2-Ge_2_Sb_2_Te_5_ calculated under compressive strains (η < 0) for electron carriers and temperatures of (a) 300 K, (b) 500 K, (c) 700 K and (d) 900 K; Figure S13: *z* direction power factor of S2-Ge_2_Sb_2_Te_5_ calculated under tensile strains (η > 0) for hole carriers and temperatures of (a) 300 K, (b) 500 K, (c) 700 K and (d) 900 K; Figure S14: Average power factor of *p*-doped S2-Ge_2_Sb_2_Te_5_ with respect to tensile strains at the temperatures of (a) 300 K, (b) 500 K, (c) 700 K and (d) 900 K; Figure S15: Average power factor of *n*-doped S2-Ge_2_Sb_2_Te_5_ with respect to compressive strains at the temperatures of (a) 300 K, (b) 500 K, (c) 700 K and (d) 900 K; Table S1: Geometrical characteristics of the octahedral cage around the Ge atom in the S1 stacking and Sb atom in the S2 stacking.
