# Supplementary material for: Electronic and Transport Properties of Strained and Unstrained Ge2Sb2Te5: A DFT Investigation
Source: Materials (Basel). 2023 Jul 15;16(14):5015. doi: 10.3390/ma16145015 (PMC10385833; doi:10.3390/ma16145015)
Supplement: Supplementary file 1 [file materials-16-05015-s001.zip › materials-2489357-supplementary.pdf]

materials

MDPI

Supplementary data

Table S1. Geometrical characteristics of the octahedral cage around the Ge atom in the S1 stacking and Sb atom in the S2 stacking. The atom labelling can be found in Fig. S7 in this supplemental section.

Parameter	Multiplicity	Value
S1 stacking		
Ge2-Te3 bond length	3	296.63 pm
Ge2-Te5 bond length	3	298.89 pm
Te3-Ge2-Te3 bond angle	3	91.084°
Te5-Ge2-Te5 bond angle	3	90.201°
Te3-Ge2-Te5 bond angle	3	179.372°
Square planar basis angle	4	90.0°
Adjacent Te3-Te5 angle in Te3-Ge2-Te5 bond angle	6	89.356°
Octahedron tilt angle w.r.t. the z-axis	/	125.120°
Octahedron face Te_i-Ge2-Te_i angles	3	60.0°
Octahedron face Te_i-Ge2-Te_j angles	3	56.627° and 60.747°
S2 stacking		
Sb2-Te3 bond length	3	300.12 pm
Sb2-Te5 bond length	3	316.34 pm
Te3-Sb2-Te3 bond angle	3	89.155°
Te5-Sb2-Te5 bond angle	3	83.503°
Te3-Sb2-Te5 bond angle	3	176.116
Square planar basis angle	4	90.0°
Adjacent Te3-Te5 angle in Te3-Sb2-Te5 bond angle	6	93.609°
Octahedron tilt angle w.r.t. the z-axis	/	129.740°
Octahedron face Te_i-Sb2-Te_i angles	3	60.0°
Octahedron face Te_i-Sb2-Te_j angles	3	55.884° and 62.058°

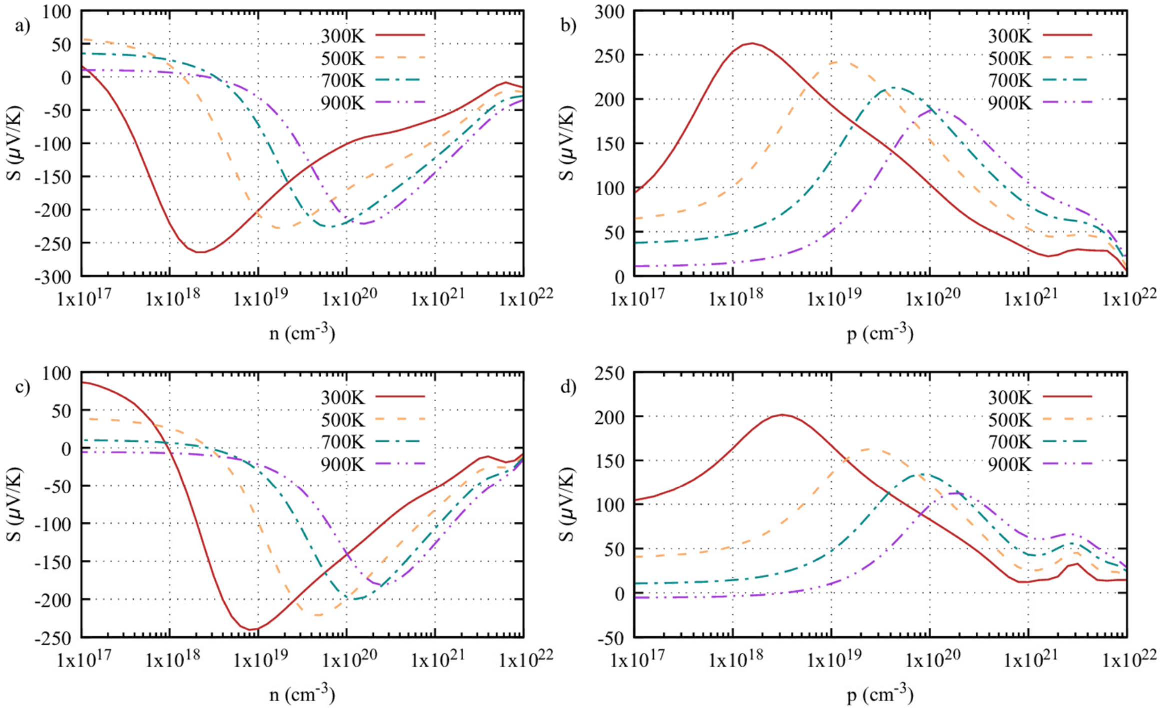

Figure S1. Seebeck coefficient in the ab plane of $Ge_2Sb_2Te_5$ at 300K, 500K, 700K and 900K for a,b) stacking 1 and c,d) stacking 2 with respect to a,c) n-type doping level and b,d) p-type doping one.

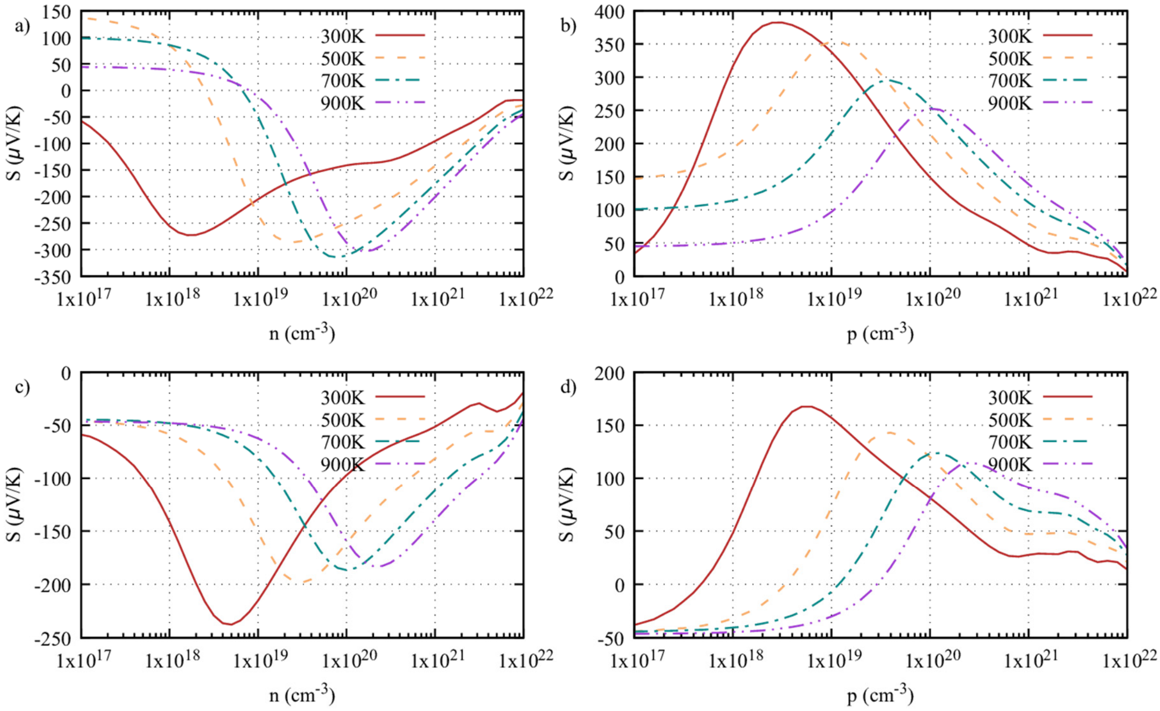

Figure S2. Seebeck coefficient in the cross plane (c axis direction) of $Ge_2Sb_2Te_5$ at 300K, 500K, 700K and 900K for a,b) stacking 1 and c,d) stacking 2 with respect to a,c) n-type doping level and b,d) p-type doping one.

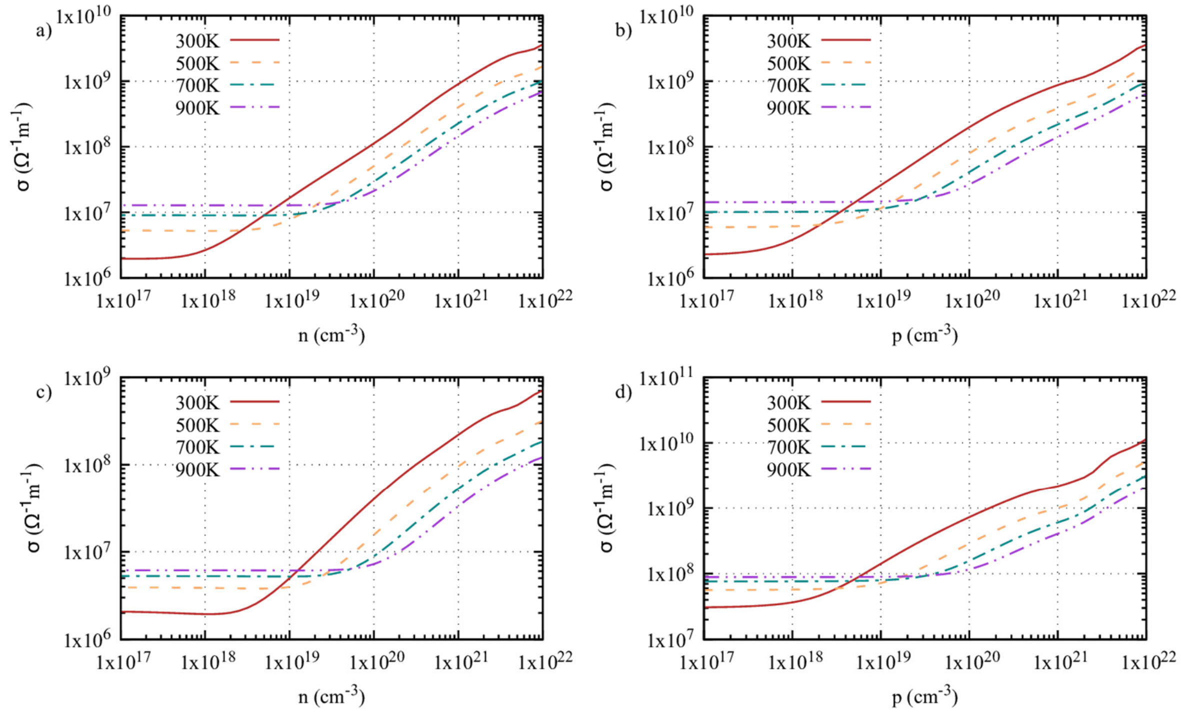

Figure S3. Electrical conductivity in the ab plane of $Ge_2Sb_2Te_5$ at 300K, 500K, 700K and 900K for a,b) stacking 1 and c,d) stacking 2 with respect to a,c) n-type doping level and b,d) p-type doping one.

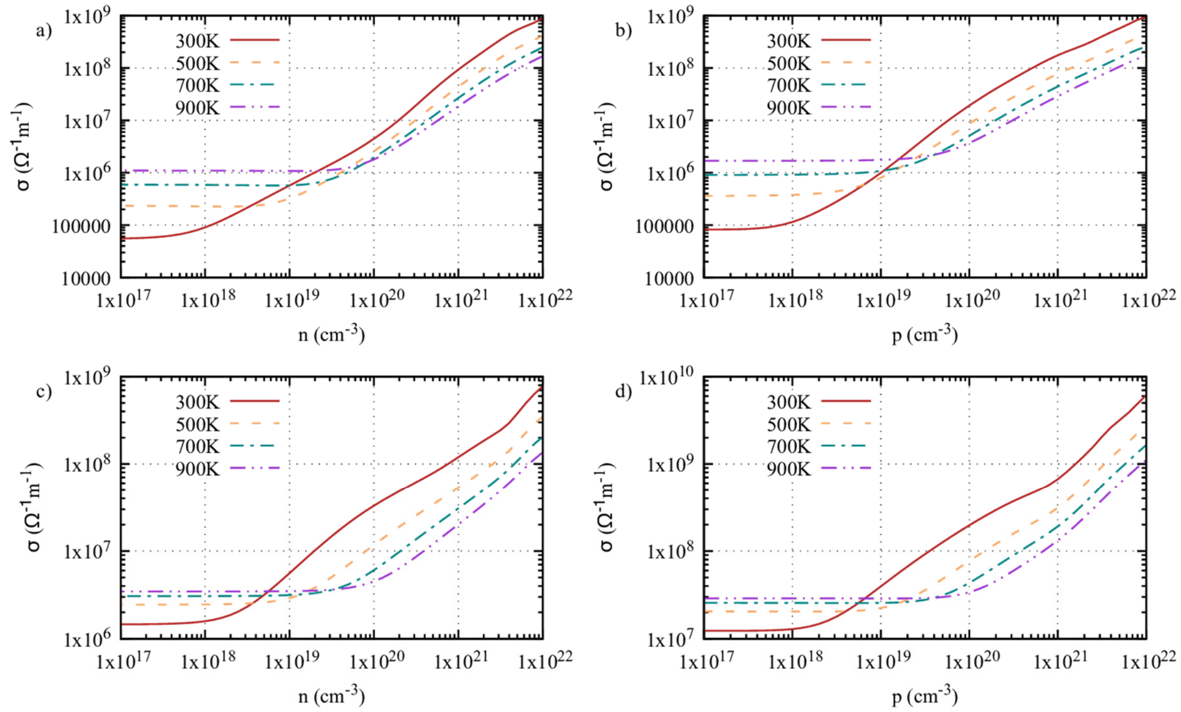

Figure S4. Electrical conductivity in the cross plane (c axis direction) of $Ge_2Sb_2Te_5$ at 300K, 500K, 700K and 900K for a,b) stacking 1 and c,d) stacking 2 with respect to a,c) n-type doping level and b,d) p-type doping one.

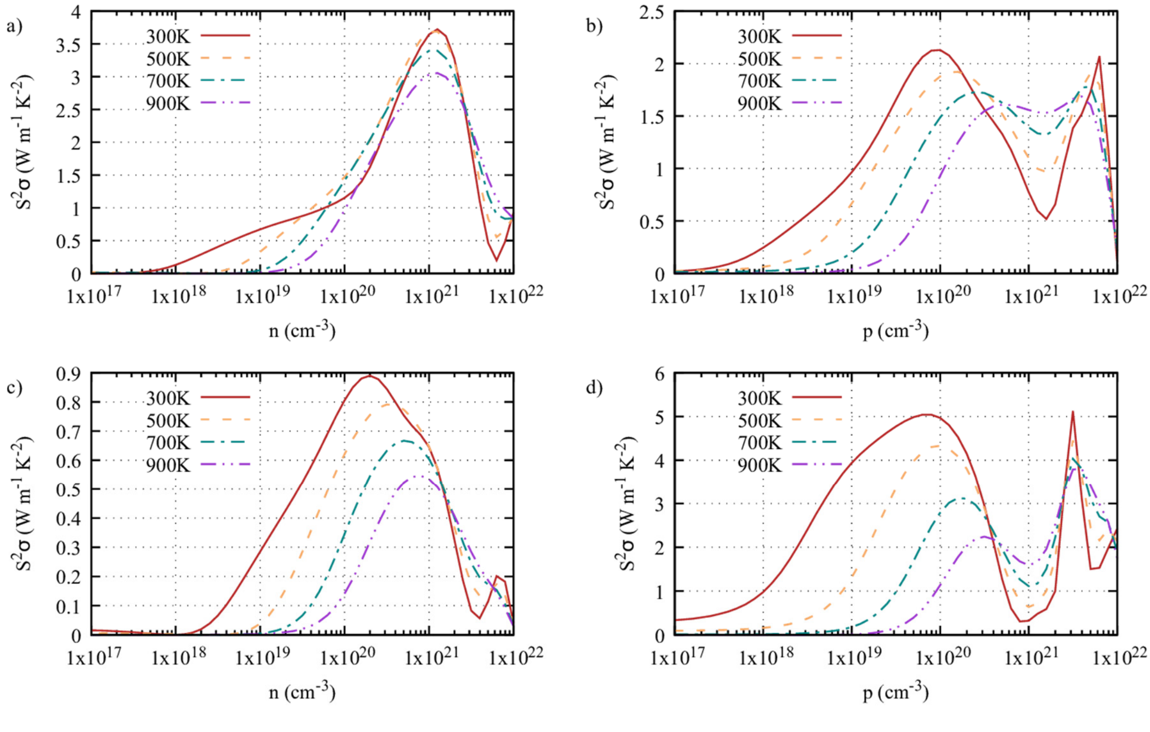

Figure S5. Power factor ($S^2\sigma$) in the ab plane of $Ge_2Sb_2Te_5$ at 300K, 500K, 700K and 900K for a,b) stacking 1 and c,d) stacking 2 with respect to a,c) n-type doping level and b,d) p-type doping one.

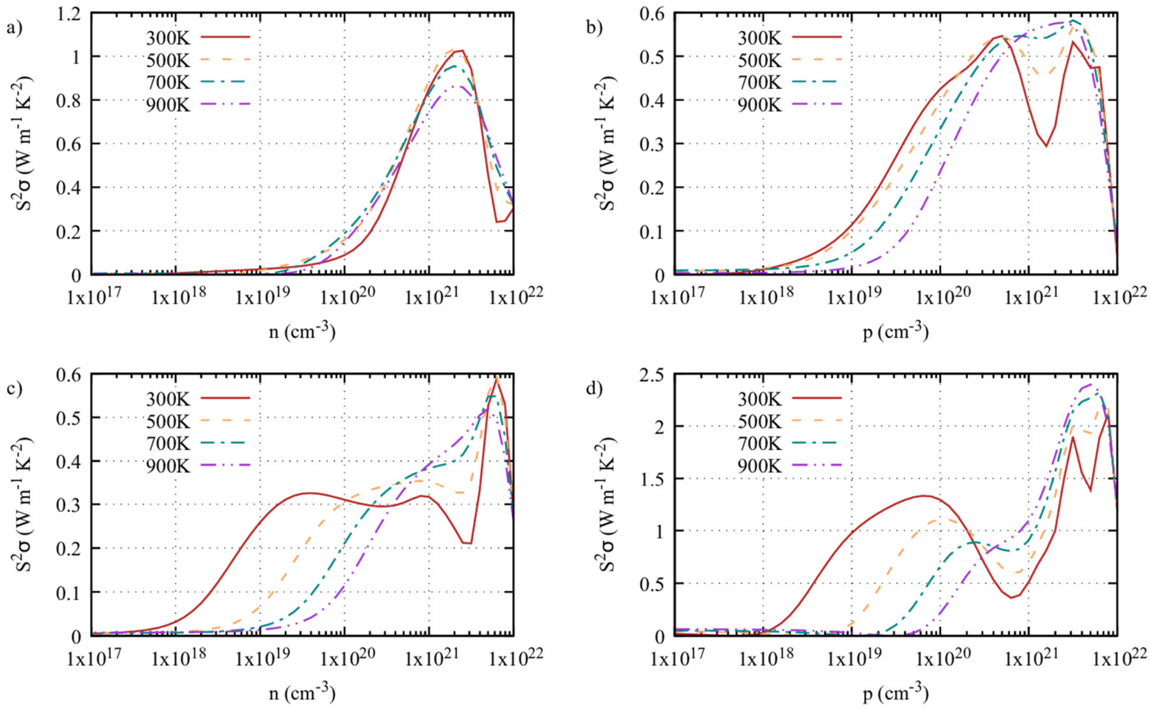

Figure S6. Power factor ($S^2\sigma$) in the cross plane (c axis direction) of $Ge_2Sb_2Te_5$ at 300K, 500K, 700K and 900K for a,b) stacking 1 and c,d) stacking 2 with respect to a,c) n-type doping level and b,d) p-type doping one.

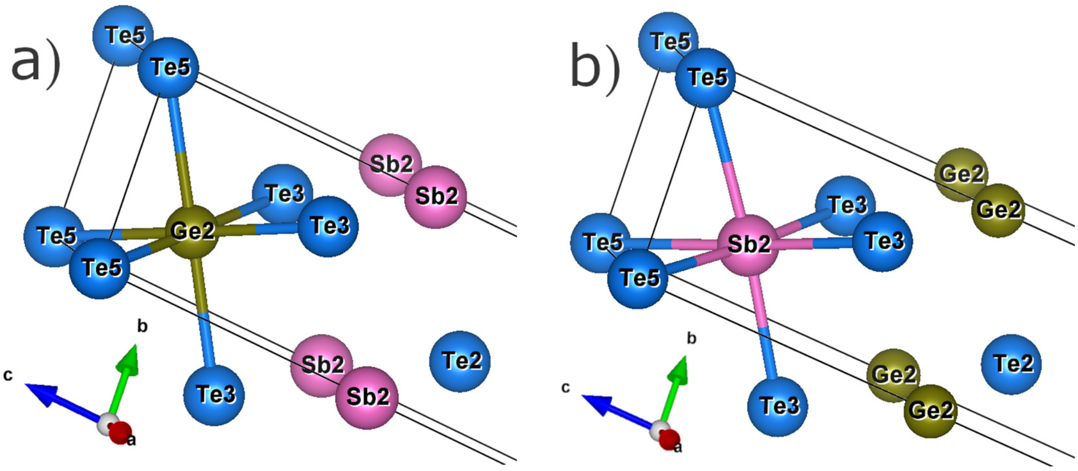

Figure S7. Deformed octahedral environment of a) Ge in S1 stacking; b) Sb in S2 stacking.

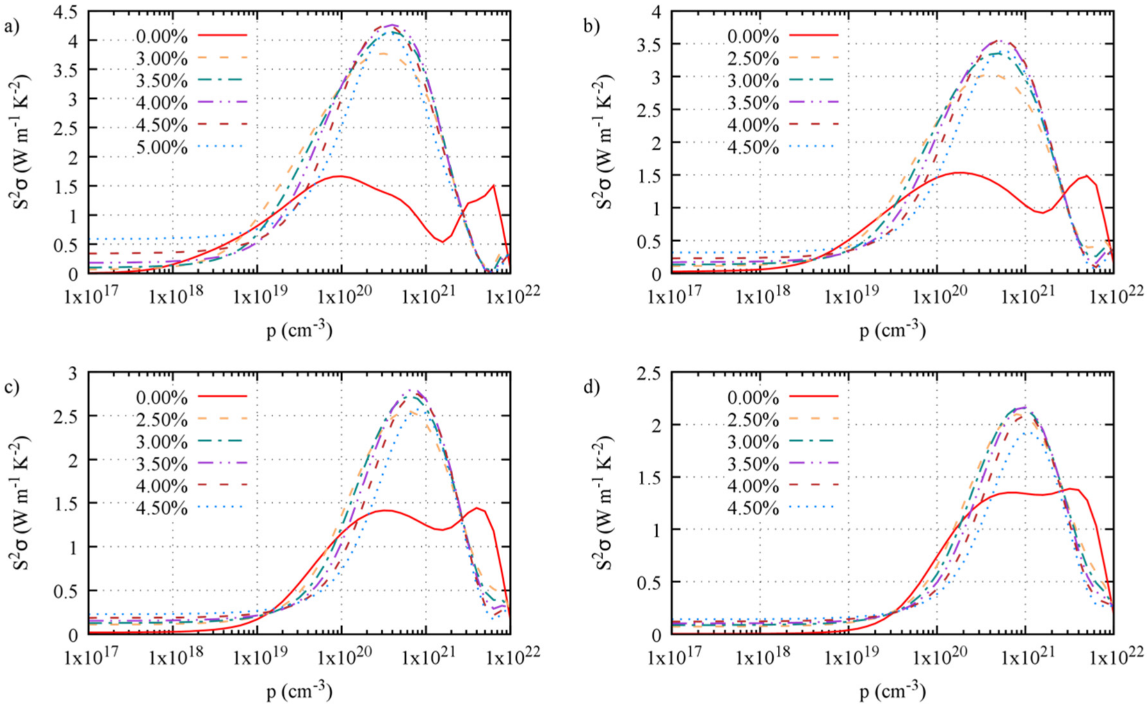

Figure S8. Average power factor of *p*-doped S1-$Ge_2Sb_2Te_5$ with respect to tensile strains at the temperatures of a) 300K, b) 500K, c) 700K, and d) 900K.

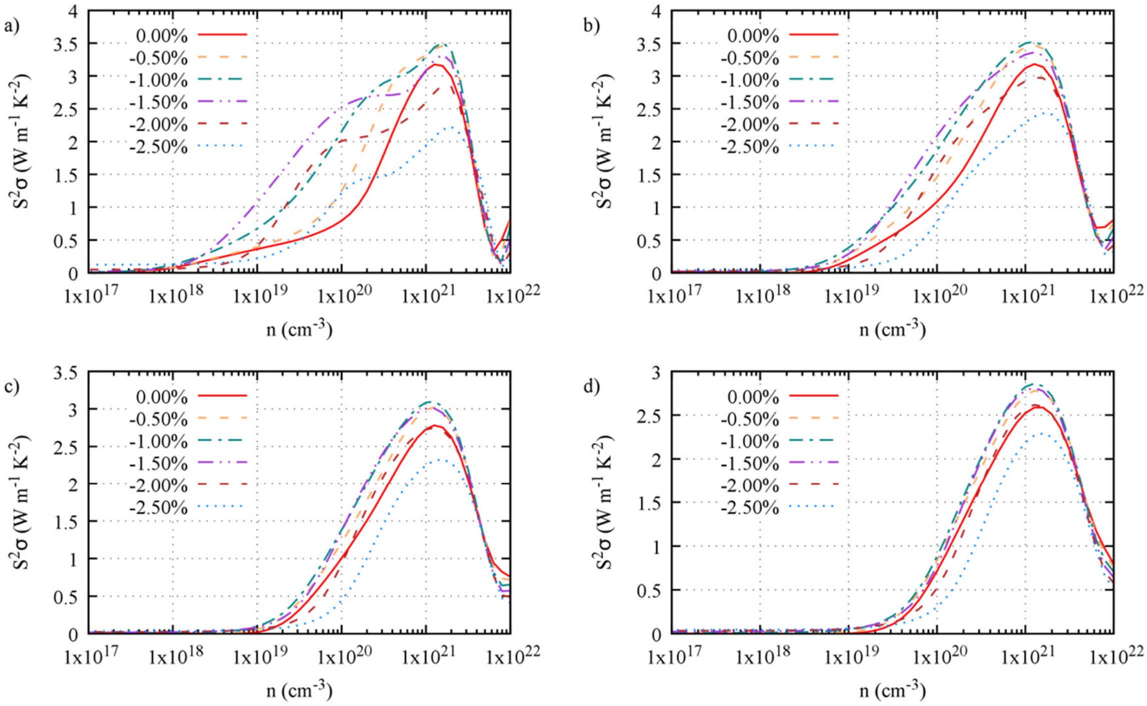

Figure S9. Average power factor of *n*-doped S1-$Ge_2Sb_2Te_5$ with respect to compressive strains at the temperatures of a) 300K, b) 500K, c) 700K, and d) 900K. .

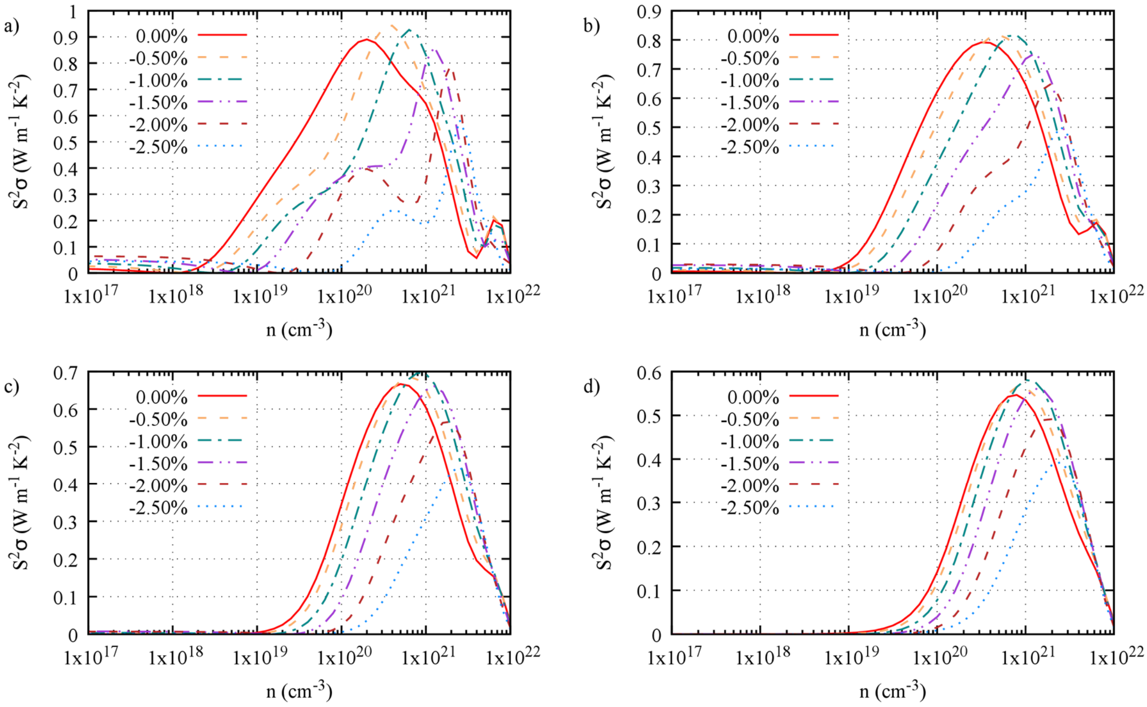

Figure S10. *ab* plane power factor of S2-$Ge_2Sb_2Te_5$ calculated under compressive strains ($\eta<0$) for electron carriers and temperatures of a) 300K, b) 500K, c) 700K and d) 900K.

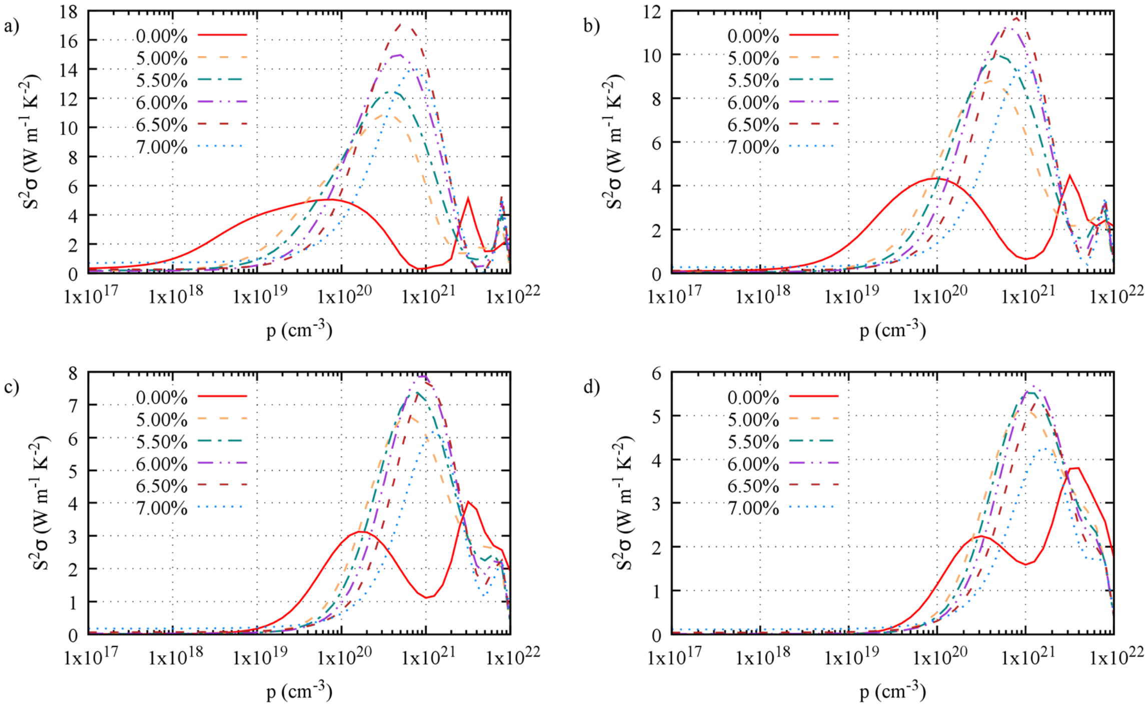

Figure S11. *ab* plane power factor of S2-$Ge_2Sb_2Te_5$ calculated under tensile strains (η>0) for hole carriers and temperatures of a) 300K, b) 500K, c) 700K and d) 900K.

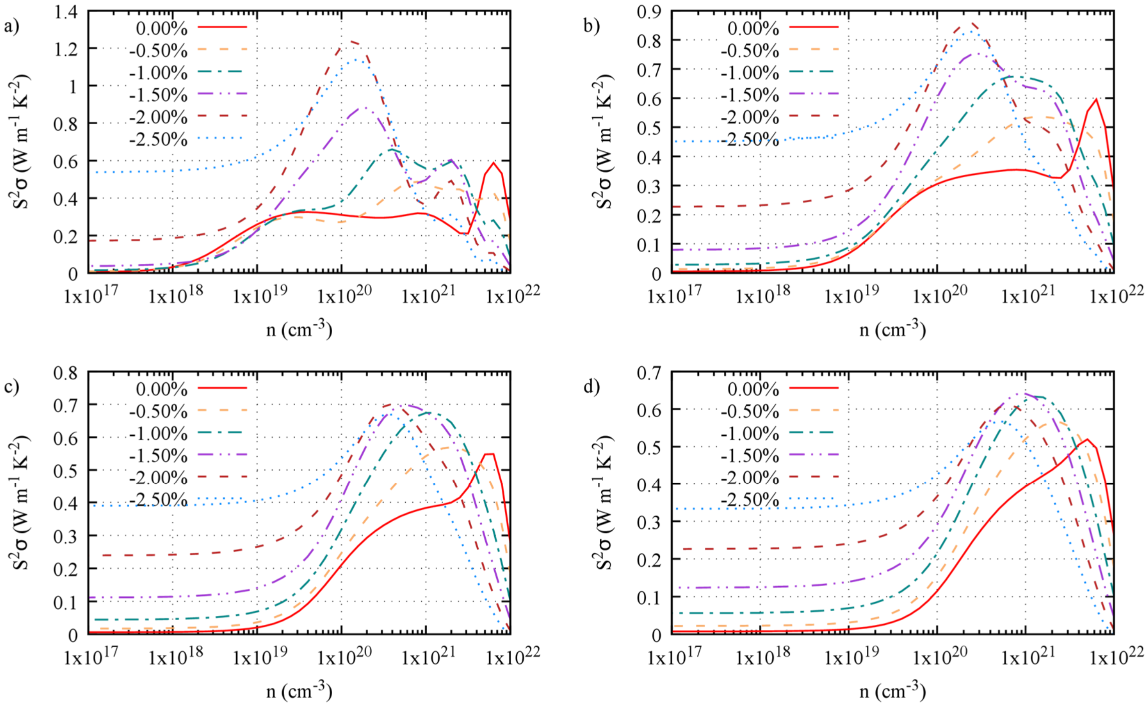

Figure S12. *z* direction power factor of S2-$Ge_2Sb_2Te_5$ calculated under compressive strains (η<0) for electron carriers and temperatures of a) 300K, b) 500K, c) 700K and d) 900K.

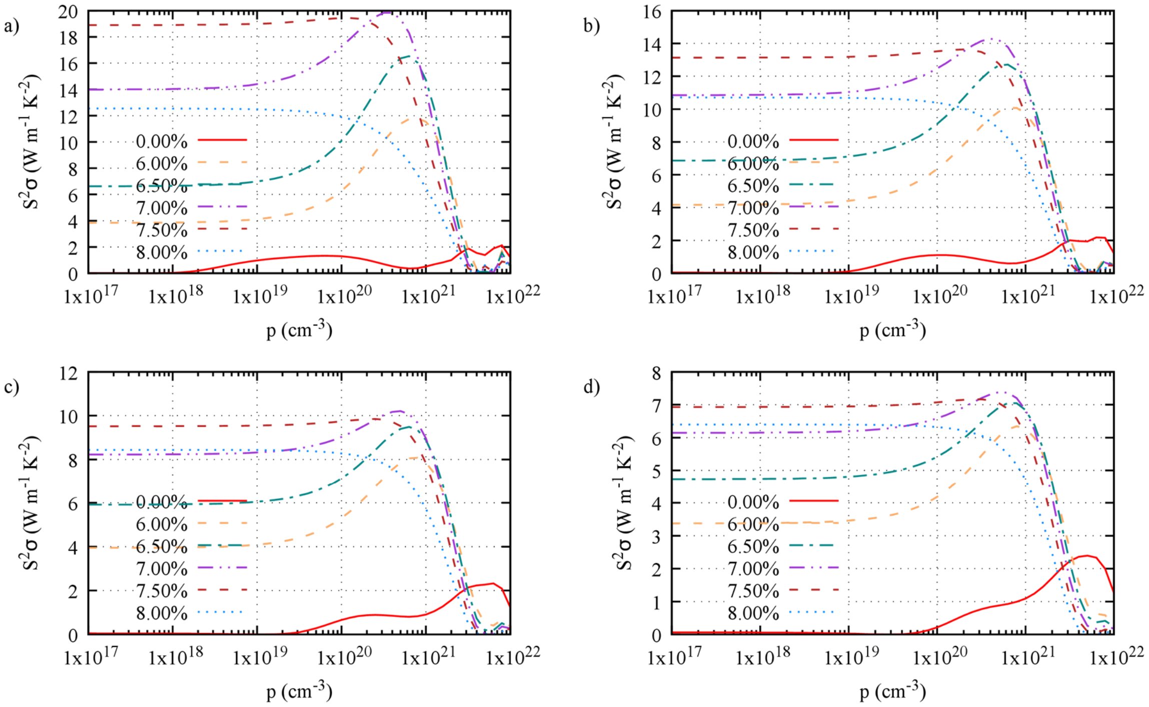

Figure S13. *z* direction power factor of S2-$Ge_2Sb_2Te_5$ calculated under tensile strains (η>0) for hole carriers and temperatures of a) 300K, b) 500K, c) 700K and d) 900K.

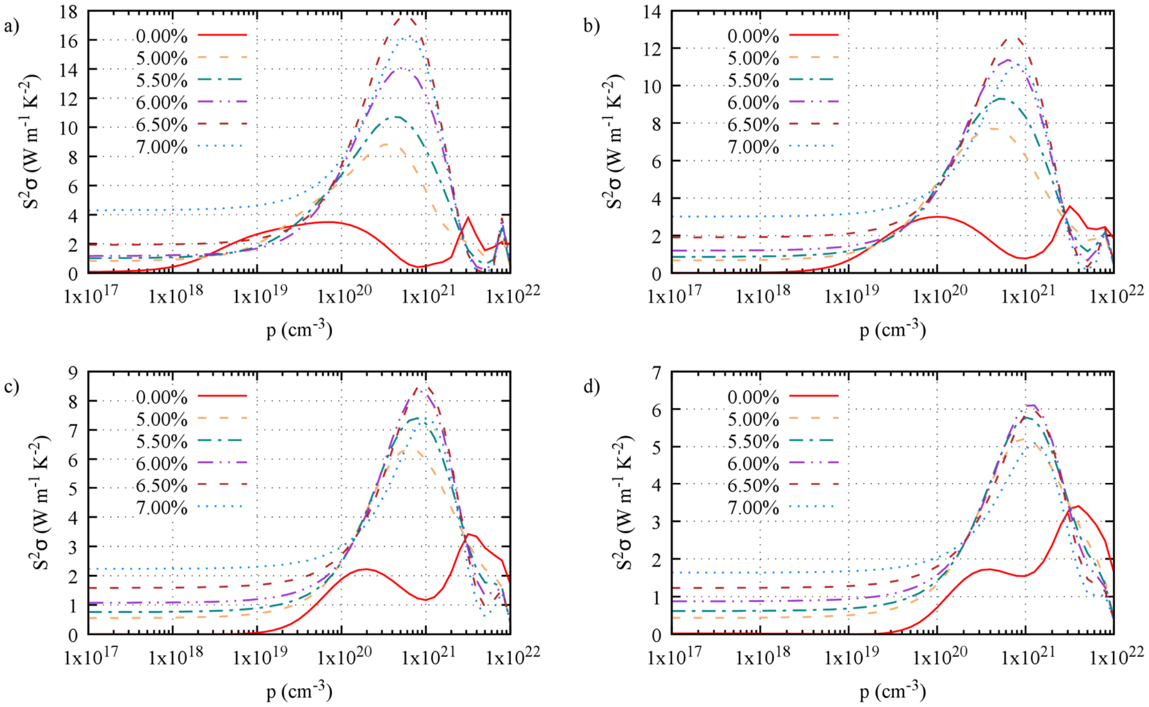

Figure S14. Average power factor of *p*-doped S2-$Ge_2Sb_2Te_5$ with respect to tensile strains at the temperatures of a) 300K, b) 500K, c) 700K, and d) 900K.

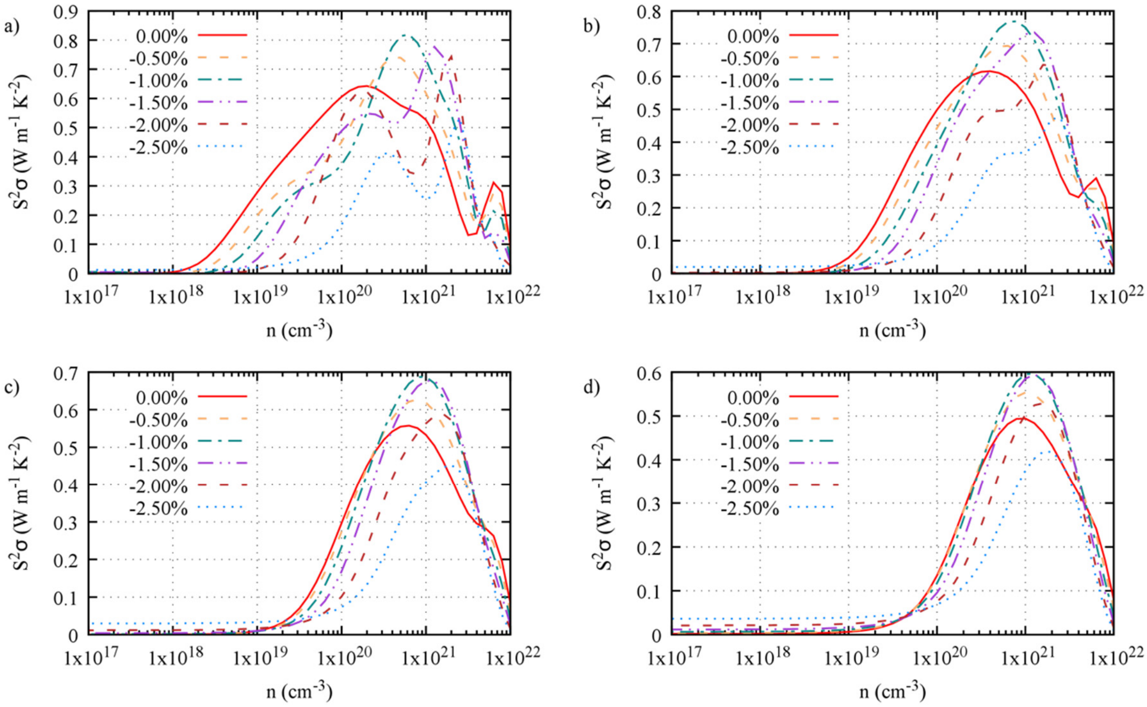

Figure S15. Average power factor of *n*-doped S2-$Ge_2Sb_2Te_5$ with respect to compressive strains at the temperatures of a) 300K, b) 500K, c) 700K, and d) 900K. .
